# Nasal Irrigation With Saline Solution for Pediatric Acute Upper Respiratory Infections: A Systematic Review

**DOI:** 10.7759/cureus.75464

**Published:** 2024-12-10

**Authors:** Márcia Cruz, Catarina Roteia, Adriana Marques Ferreira, Roberta Barros, Hugo Sevivas, Tânia Gomes, Andreia Silva Bandeira

**Affiliations:** 1 Family Medicine, USF Physis, Unidade Local de Saúde de Alto Ave, Guimarães, PRT; 2 Family Medicine, USF Saúde Oeste, Unidade Local de Saúde de Braga, Braga, PRT; 3 Family Medicine, USF Ruães, Unidade Local de Saúde de Braga, Braga, PRT; 4 Family Medicine, USF Gualtar, Unidade Local de Saúde de Braga, Braga, PRT; 5 Family Medicine, USF São Vítor, Unidade Local de Saúde de Braga, Braga, PRT

**Keywords:** acute upper respiratory tract infections, adjunct therapy, nasal irrigation, pediatric infection management, pediatrics, saline nasal lavage, symptom relief

## Abstract

Acute upper respiratory tract infections (URTIs) are defined as infectious diseases confined anatomically to the upper respiratory tract, with a duration of up to 28 days. Treatment for URTIs in pediatrics typically involves antipyretics and decongestants and, at times, antibiotics, despite most infections being viral. Nasal irrigation with saline solution is frequently used as an adjunct treatment for URTI symptoms. Currently, there is no consensus on whether nasal irrigation with saline offers significant benefits in reducing the severity of URTI symptoms. This systematic review aims to assess the scientific evidence supporting nasal irrigation with saline as a recommendation to alleviate symptom severity in pediatric URTI cases. We conducted a systematic literature search in PubMed, Cochrane Library, Medline, and Scopus databases, covering the period from January 2010 to May 2024 in Portuguese, Spanish, and English. MeSH terms used were: (((pediatrics) OR (children) OR (infant) OR (adolescent) OR (child)) AND ((isotonic saline solution) OR (nasal douching) OR (nasal saline irrigation) OR (nasal lavage fluid)) AND ((respiratory disease) OR (upper respiratory tract infection))). Only randomized controlled trials were included, and we assessed the risk of bias using the Cochrane risk-of-bias tool (ROBIS 2.0). The Strength of Recommendation Taxonomy (SORT) was applied to determine the level of evidence and strength of recommendation. The initial search yielded 158 articles, of which only four met the criteria for inclusion in this systematic review. All studies evaluated whether saline nasal irrigation, with or without additional treatments, contributes to symptom improvement in pediatric URTIs. Notably, some studies reported symptom relief and even faster recovery times with its use. Based on our analysis, nasal irrigation with saline solution may reduce symptom severity in children with URTIs, with a level of evidence of 2 and a recommendation strength of B. Further research with more robust methodologies is needed to confirm these findings.

## Introduction and background

Acute upper respiratory tract infections (AURTIs) are defined as infectious conditions anatomically limited to the upper respiratory tract, including the nose, throat, ears, and sinuses, and have a typical duration of up to 28 days [1]. They represent one of the most common illnesses affecting pediatric populations worldwide, accounting for a significant portion of outpatient visits and antibiotic prescriptions. The high prevalence of these infections not only burdens healthcare systems but also leads to substantial parental absenteeism from work and decreased quality of life for affected children [2]. These infections are primarily caused by a variety of respiratory viruses, with rhinovirus being the most common pathogen. Such viruses are predominantly found in nasal secretions and are easily transmitted via actions such as sneezing, coughing, or nose-blowing. The common cold, a frequent manifestation of AURTIs, typically presents with symptoms such as fever, cough, rhinorrhea, nasal congestion, sore throat, headache, and generalized muscle pain or myalgia [1,2].

Management of AURTIs in pediatric populations generally focuses on symptomatic relief. This often involves the use of antipyretics to manage fever and decongestants for nasal symptoms. However, antibiotics are occasionally prescribed despite the lack of evidence supporting their effectiveness, as the majority of these infections are viral rather than bacterial in origin [3].

Saline nasal irrigation, a non-pharmacological intervention, has gained increasing attention due to its simplicity, safety, and potential benefits in reducing symptom severity and duration, rather than OTC medications or topical aromatic therapies. In infants, topical saline can be applied with saline nose drops or a bulb syringe. In older children, either a saline nasal spray or saline nasal irrigation (squeeze bottle, neti pot, or nasal douche) can be used. Saline irrigants must be prepared from sterile or bottled water. This technique works by mechanically clearing mucus, pathogens, and allergens from the nasal passages, potentially enhancing mucociliary clearance and reducing inflammation [4,5]. While the use of saline nasal irrigation has been widely recommended for chronic upper airway conditions, its benefits in the context of AURTIs are less well established. Currently, there is no consensus on whether nasal irrigation significantly reduces the severity or duration of AURTI symptoms, although healthcare professionals frequently suggest it as an adjunctive therapy for managing these conditions [5].

This review seeks to systematically evaluate the evidence supporting the use of saline nasal irrigation in the management of pediatric AURTIs, aiming to provide clarity on its therapeutic value and practical application.

## Review

Methods

To conduct this systematic review, the authors searched the PubMed, Cochrane Library, Medline, and Scopus databases in May 2024. The review adhered to the Preferred Reporting Items for Systematic Reviews and Meta-Analyses (PRISMA) guidelines [6], which provide a structured framework for conducting high-quality systematic reviews. To formulate the research question, the authors employed the PICO framework, which considers the Population, Intervention, Comparison, and Outcomes as key elements of the investigation. The inclusion criteria for this work were articles written in Portuguese, Spanish, or English, studies published between January 2010 and May 2024, and randomized controlled trials (RCTs) that adhered to the predefined PICO framework. As for the exclusion criteria, the authors decided on studies that fell outside the inclusion criteria, such as meta-analyses, systematic reviews, cohort studies, case-control studies, cross-sectional studies, laboratory (in vitro) research, and animal studies; case reports, opinion pieces, letters to the editor, and articles unavailable in full-text format; and duplicated or redundant publications. The inclusion criteria were deliberately focused on RCTs to ensure the highest level of evidence. Language restrictions (Portuguese, Spanish, and English) were applied to balance the feasibility of the review with the breadth of coverage. Exclusion criteria were designed to filter out lower-quality evidence, such as observational studies, case reports, and opinion pieces while minimizing bias from studies unavailable in full-text format.

Search Strategy

The authors employed a structured query using Boolean operators to combine keywords such as pediatrics, children, isotonic saline solution, nasal irrigation, and upper respiratory tract infection. The final search string was as follows: (((pediatrics) OR (children) OR (infant) OR (adolescent)) AND ((isotonic saline solution) OR (nasal irrigation)) AND ((respiratory disease) OR (upper respiratory tract infection))).

Study Selection

**Figure 1:**
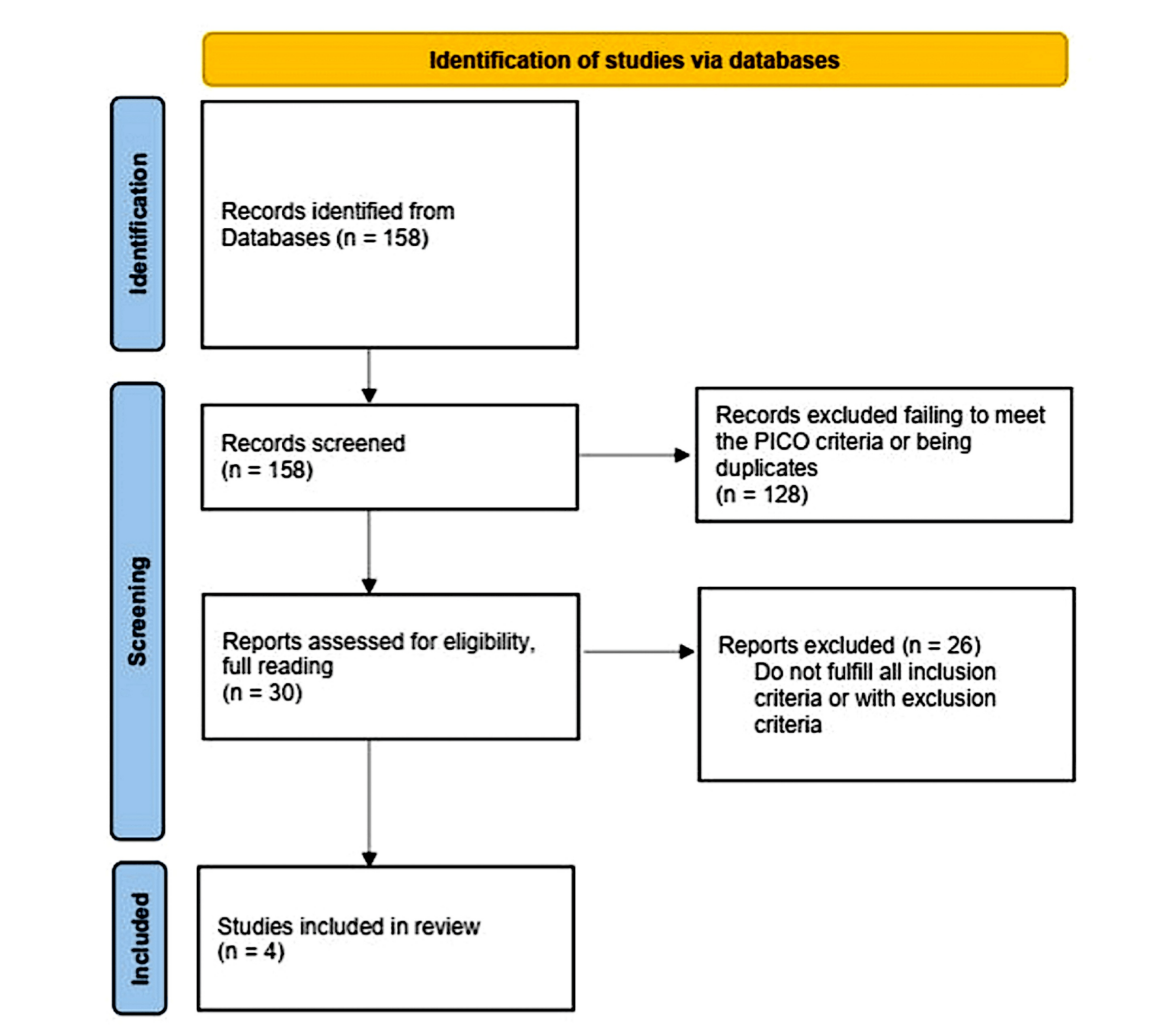
PRISMA diagram PRISMA: Preferred Reporting Items for Systematic Reviews and Meta-Analyses [6]

The process of article inclusion is depicted in the PRISMA diagram (Figure 1). The database search identified 158 articles, regardless of article type. After title screening, 128 articles were excluded for either failing to meet the PICO criteria (as outlined above) or being duplicates. The remaining 30 articles were reviewed in full, resulting in four studies that met all the inclusion criteria.

Data Extraction and Analysis

Data extraction was independently performed by two authors to reduce the risk of bias. Extracted information included study design, population demographics, interventions, outcomes, results, and limitations. Discrepancies between reviewers were resolved through discussion or consultation with a third author.

Risk of Bias and Evidence Grading

The risk of bias in the included studies was evaluated using the Revised Cochrane Risk-of-Bias Tool for Randomized Trials (RoB 2.0) [7]. Additionally, the Strength of Recommendation Taxonomy (SORT), developed by the American Academy of Family Physicians [8], was applied to grade the level of evidence and strength of recommendations.

Results

The authors independently evaluated each article and developed a results table, including study reference, study type, sample size, intervention, outcomes, results, limitations, risk of bias, and level of evidence of the study. The narrative review was written following data entry and analysis of Table 1.

**Table 1:** Study results G1/G2/Group A, B, C: study groups IG: intervention group; CG: control group; PRQLQ: Pediatric Rhinoconjunctivitis Quality of Life Questionnaire

Reference and Study Type	Population	Intervention	Outcomes	Results	Limitations	RoB 2	Level of Evidence
Alexandrino AS et al. [9] (2018) Randomized controlled trial	n = 75; Children under 3 years old with acute upper respiratory infection (URTI) symptoms, divided into: IG (n = 37) and CG (n = 38)	IG: Isotonic saline nasal irrigation followed by forced nasal inspiration for 30 minutes. Baseline protocol included otoscopy, tympanometry, and nasal auscultation. CG: No intervention.	Evaluate the benefits of nasal irrigation on nasal obstruction, middle ear pressure peak, and tympanic membrane compliance, assessed via tympanometry and nasal auscultation.	Significant improvements in nasal obstruction and middle ear pressure in IG compared to CG (*p* = 0.042). There were no significant differences in tympanic membrane compliance in either group.	Small sample size; short intervention and follow-up periods; potential variability in intervention execution.	Moderate (some concerns)	2
Khoshdel A et al. [10](2014) Randomized, double-blind, controlled trial	n = 100; Children aged 4–15 years with acute sinusitis, divided into: G1 (n = 50) and G2 (n = 50)	G1: Amoxicillin (80 mg/kg/day) in three doses for 14 days, with saline nasal irrigation (0.9%) 1–3 times/day for 5 days and phenylephrine (0.25%) 2–3 times/day for 2 days. G2: Saline nasal irrigation (0.9%) and phenylephrine (0.25%) without antibiotics.	Compare the efficacy of amoxicillin combined with saline nasal irrigation versus saline irrigation alone in resolving acute sinusitis symptoms, evaluated on days 3, 14, 21, and 28.	G1 showed faster symptom resolution by day 3 (*p* < 0.001). By day 14, cure rates were similar (97.5% in G1 vs. 95% in G2). No significant differences in outcomes by days 21 and 28 (*p* > 0.05).	Small sample size; subjective or non-validated methods; differences between clinical and outpatient settings; short follow-up period.	Moderate (some concerns)	2
Köksal T et al. [11] (2016) Prospective, double-blind, randomized trial	n = 125; Children under 2 years old with acute URTI. Mean age: 9.0 ± 3.9 months; 65 boys (59.6%), 44 girls (40.4%)	Group A (n = 38): Hypertonic saline (2.3%) for nasal irrigation. Group B (n = 36): Isotonic saline (0.9%) for nasal irrigation. Group C (n = 35): No nasal irrigation.	Compare the efficacy of hypertonic saline, isotonic saline, and no intervention on nasal congestion, rhinorrhea, epistaxis, cough, fatigue, and sleep quality.	Significant symptom improvement and better sleep quality in Groups A and B compared to Group C (*p* < 0.05). No major differences between Groups A and B (*p* > 0.05).	Small sample size; dependence on caregiver adherence; variability in administration methods; subjective measures; short follow-up period.	Moderate (some concerns)	2
Ragab A et al. [12] (2015) Prospective, double-blind, placebo-controlled trial	n = 106; children with uncomplicated acute rhinosinusitis, divided into: G1 (n = 41) and G2 (n = 43)	G1: Amoxicillin (100 mg/kg/day) and saline nasal irrigation (0.9%) 1–3 times/day for 14 days. G2: Placebo and saline nasal irrigation (0.9%) 1–3 times/day for 14 days.	Evaluate clinical improvement using nasal symptom scores, quality of life (PRQLQ), bacteriological and cytological responses in the middle meatus (MM), and adverse effects.	Clinical cure observed in 83.9% of G1 and 71% of G2 (*p* = 0.22). No significant difference in PRQLQ scores (*p* = 0.06). Fewer adverse effects in G2 (*p* = 0.005). MM neutrophil reduction significant in G1 (*p* = 0.004).	Lack of imaging evaluation (CT scans); short follow-up period.	Moderate (some concerns)	2
Strength of recommendation: B							

Alexandrino et al. [9] investigated whether nasal irrigation provides benefits for nasal obstruction, middle ear pressure peak, and tympanic membrane compliance, as assessed through nasal auscultation and tympanometry. The study included 75 children under the age of three, of both genders, presenting with AURTI symptoms. The participants were divided into two groups: the intervention group (IG) that underwent baseline otoscopy and tympanometry, followed by isotonic saline nasal irrigation, forced nasal inspiration for 30 minutes, and nasal auscultation, and the control group (CG) that underwent baseline otoscopy and tympanometry without further intervention. Following the interventions, all participants repeated the baseline protocol. The results showed significant differences between the groups, with a higher frequency of children in the IG exhibiting unobstructed nasal sounds after nasal irrigation compared to the CG (unobstructed sound: IG: 66.7% vs. CG: 31.6%; p = 0.042). Intragroup analysis revealed an improvement in the middle ear pressure peak bilaterally in the IG immediately after the intervention, a change not observed in the CG.

Khoshdel et al. [10] aimed to compare the efficacy of amoxicillin combined with nasal irrigation versus nasal irrigation alone in resolving acute sinusitis symptoms in children, assessed on days 3, 14, 21, and 28 of treatment. Clinical symptoms evaluated included nasal discharge, nasal congestion, headache, and cough, with complete resolution defined as the absence of all sinusitis-related signs and symptoms. The study involved 100 children aged 4-15 years with acute sinusitis, divided into two groups. Group 1 (G1, n=50) received oral amoxicillin (80 mg/kg/day in three divided doses) for 14 days, along with 0.9% saline nasal irrigation (one to three times daily for five days) and 0.25% phenylephrine (two to three times daily for two days), and Group 2 (G2, n=50) was treated with 0.9% saline nasal irrigation (one to three times daily for five days) and 0.25% phenylephrine (two to three times daily for two days) without antibiotics.

On day three of treatment, 34 children (85%) in G1 were classified as cured compared to 15 children (37.5%) in G2 (p < 0.001). By day 14, the cure rate was 97.5% in G1 and 95% in G2. By days 21 and 28, all participants were fully cured without any relapse. A statistically significant difference was observed only on day three, with G1 showing greater symptom resolution and clinical improvement (p < 0.001). On subsequent follow-up days, no significant difference in the healing process was noted between the two groups (p > 0.05).

Köksal et al. [11] compared the efficacy of isotonic saline, hypertonic saline, and no intervention in alleviating clinical symptoms, such as nasal congestion, rhinorrhea, epistaxis, cough, fatigue, and sleep disturbance, associated with AURTIs. The study included 125 children under two years of age with AURTIs, divided into three groups: Group A (n=38) was treated with hypertonic saline (2.3%) for nasal irrigation; Group B (n=36) was treated with isotonic saline (0.9%) for nasal irrigation; and Group C (n=35) had no nasal irrigation performed. The results demonstrated clinical improvement and better sleep quality in children receiving saline irrigation (both hypertonic and isotonic) compared to those without irrigation. This indicates that saline solutions can be beneficial as adjuvants in managing URTIs in children.

In the study by Ragab et al. [12], 106 children with uncomplicated acute rhinosinusitis were divided into two groups to evaluate clinical improvement using nasal symptom scores, quality of life through the Pediatric Rhinoconjunctivitis Quality of Life Questionnaire (PRQLQ), bacteriological and cytological responses in the middle meatus (MM), and adverse effects. Group 1 (G1, n=41) was treated with amoxicillin (100 mg/kg/day) and 0.9% saline nasal irrigation (one to three times daily) for 14 days, and Group 2 (G2, n=43) was treated with a placebo and 0.9% saline nasal irrigation (one to three times daily) for 14 days. The clinical cure rates were 83.9% in G1 compared to 71% in G2, with no statistically significant difference (p = 0.22). The mean total PRQLQ scores also showed no significant differences after two weeks (p = 0.06). However, on day seven, MM neutrophil counts significantly decreased in G1 compared to G2 (p = 0.004). By day 14, there were no significant differences in MM cytological content between the groups (p = 0.07). Adverse effects such as diarrhea, abdominal pain, and nausea were more common in G1 than in G2 (p = 0.005). The authors concluded that saline nasal irrigation alone provides comparable clinical, bacteriological, and cytological efficacy to amoxicillin with a superior safety profile in children with uncomplicated acute rhinosinusitis after 14 days of treatment.

The studies reviewed consistently demonstrated the effectiveness of saline nasal irrigation in providing symptom relief for pediatric AURTIs. Significant reductions in nasal congestion and improvements in respiratory function were observed across all studies, emphasizing the intervention's role in alleviating discomfort associated with these conditions. Notably, while isotonic saline proved effective, some studies indicated that hypertonic saline offered marginally superior outcomes, particularly in addressing nasal obstruction. Additionally, the safety profile of saline nasal irrigation was robust, with no major adverse events reported, making it a well-tolerated option for young children. However, variability in outcomes was noted, largely influenced by adherence to irrigation protocols. This highlights the critical importance of proper technique and active caregiver involvement to optimize the intervention's efficacy.

Discussion

This systematic review aimed to evaluate the evidence supporting the use of saline nasal irrigation as an adjunctive treatment for AURTIs in pediatric populations. The findings from the selected studies consistently demonstrated symptomatic benefits, indicating that nasal irrigation can play a valuable role in managing AURTIs. Despite this, several important considerations and limitations must be addressed.

The studies reviewed highlight the clinical utility of saline nasal irrigation in reducing symptoms associated with AURTIs. Alexandrino et al. [9] demonstrated immediate improvements in nasal obstruction and middle ear pressure in children who underwent saline nasal irrigation compared to controls. Similarly, Köksal et al. [11] reported significant symptomatic relief and improved sleep quality in children treated with isotonic or hypertonic saline solutions compared to no intervention. These findings suggest that saline nasal irrigation provides rapid and noticeable relief, which can be particularly valuable in alleviating discomfort in pediatric patients.

In cases of acute sinusitis, the studies by Khoshdel et al. [10] and Ragab et al. [12] further supported the efficacy of saline nasal irrigation. While Khoshdel et al. [10] observed faster symptom resolution when nasal irrigation was combined with amoxicillin, Ragab et al. [12] demonstrated that saline nasal irrigation alone was equally effective as amoxicillin in terms of clinical and bacteriological outcomes, with fewer adverse effects. These results underscore the potential of saline nasal irrigation as a standalone treatment for uncomplicated AURTIs, particularly when antibiotic use is unnecessary or contraindicated.

Despite the consistent benefits observed, the studies included in this review are not without limitations. A common concern across all studies is the relatively small sample size, which may limit the generalizability of the findings. Larger-scale studies are needed to confirm these results and provide more robust evidence to guide clinical recommendations.

Another limitation relates to the variability in adherence to nasal irrigation techniques. Proper execution of the technique is critical for achieving optimal outcomes, as highlighted in several studies and supported by additional literature. Poor technique or inconsistent adherence, whether due to lack of understanding or difficulty performing the procedure, can significantly impact results. This issue is not only a limitation in the studies but also reflects real-world challenges faced by clinicians when recommending nasal irrigation to parents or caregivers. Variations in the method, frequency, and volume of saline used can further contribute to inconsistencies in outcomes. Another variable could be the concentration of the solution or the delivery method, since the primary mechanism by which saline nasal irrigation provides relief is through mechanical clearance of mucus and pathogens. Enhanced mucociliary transport, reduced inflammatory mediators, and maintenance of nasal epithelial hydration contribute to symptom relief. However, the degree of benefit may depend on factors such as solution concentration (isotonic vs. hypertonic) and delivery method (e.g., sprays vs. neti pots).

The follow-up duration in most studies was relatively short, which restricted the ability to assess long-term efficacy and potential recurrence of symptoms. For instance, while Alexandrino et al. [9] and Köksal et al. [11] reported immediate improvements, neither study investigated whether these benefits persisted beyond the intervention period. Longer follow-up periods would provide valuable insights into the sustained effectiveness of saline nasal irrigation.

The user-dependent nature of nasal irrigation presents an additional challenge. As noted by the authors and supported by existing literature, the success of nasal irrigation relies heavily on proper technique. Inconsistent execution not only undermines the efficacy of the treatment but may also contribute to disparities in outcomes across different populations. This raises an important question about the practicality of nasal irrigation as a widely recommended intervention. Ensuring adequate caregiver education and adherence to best practices may help address this issue, but it also highlights the need for further research into simplified or standardized approaches to nasal irrigation.

One of the most significant advantages of saline nasal irrigation is its safety profile. Across all studies, no serious adverse effects were reported, and the few minor side effects observed, such as gastrointestinal complaints in Ragab et al. [12], were associated with concurrent antibiotic use rather than the nasal irrigation itself. This makes saline nasal irrigation an attractive option for managing AURTIs, particularly in pediatric patients, where minimizing drug exposure is a priority.

The findings of this review suggest that saline nasal irrigation is a valuable adjunctive therapy for AURTIs in children, offering a safe and effective method for reducing symptom severity and improving quality of life. However, to maximize its potential, further research is needed to address the identified limitations and provide more comprehensive guidance for clinical practice.

Future studies should focus on addressing the methodological limitations of existing research. Larger, multicenter RCTs with diverse populations would provide more generalizable evidence. Additionally, standardized protocols for nasal irrigation, detailing the volume, frequency, and type of saline solution, should be developed and evaluated to ensure consistency across studies and in clinical practice.

Longer follow-up periods are also essential to assess the durability of symptomatic improvements and the potential impact of saline nasal irrigation on recurrence rates and long-term outcomes. Finally, further exploration of caregiver training programs and their impact on adherence and technique effectiveness would help bridge the gap between clinical research and real-world application.

In summary, while the current evidence supports the use of saline nasal irrigation as an effective adjunctive treatment for AURTIs in children, limitations in study design, sample size, and follow-up duration warrant further investigation. Despite these challenges, the safety, accessibility, and observed benefits of saline nasal irrigation highlight its potential as a valuable addition to pediatric AURTI management strategies. The application of the SORT [8] framework to the findings of this review assigns a Level 2 evidence grade and strength of recommendation B, supporting its consideration in routine clinical practice.

## Conclusions

This review highlights the potential benefits of saline nasal irrigation as an adjunctive treatment for pediatric AURTIs. Key findings from the included studies consistently demonstrated that saline nasal irrigation significantly alleviates symptoms such as nasal congestion, rhinorrhea, and postnasal drip. These improvements were often observed within a short duration and were associated with enhanced quality of life and minimal adverse effects. Importantly, saline nasal irrigation proved to be an effective standalone intervention for uncomplicated conditions, such as acute rhinosinusitis, with outcomes comparable to antibiotic use but with fewer side effects. However, its success is user-dependent, requiring proper technique and adherence, which may vary in clinical and real-world settings.

Despite the small sample sizes and short follow-up periods across studies, the evidence suggests that saline nasal irrigation is a safe, accessible, and effective intervention for managing pediatric AURTIs. Its integration into clinical practice could reduce the reliance on antibiotics, thereby addressing concerns about antimicrobial resistance while improving patient outcomes. Future research should focus on larger-scale studies with standardized protocols to further validate these findings and optimize implementation.
